# Laminin N‐terminus α31 expression during development is lethal and causes widespread tissue‐specific defects in a transgenic mouse model

**DOI:** 10.1096/fj.202002588RRR

**Published:** 2022-06-01

**Authors:** Conor J. Sugden, Valentina Iorio, Lee D. Troughton, Ke Liu, Mychel R. P. T. Morais, Rachel Lennon, George Bou‐Gharios, Kevin J. Hamill

**Affiliations:** ^1^ Institute of Life Course and Medical Sciences University of Liverpool Liverpool UK; ^2^ Department of Cell and Molecular Physiology Stritch School of Medicine Loyola University Chicago Maywood Illinois USA; ^3^ Wellcome Centre for Cell‐Matrix Research Division of Cell‐Matrix Biology and Regenerative Medicine The University of Manchester Manchester UK

**Keywords:** basement membrane, development, laminin, netrin

## Abstract

Laminins (LMs) are essential components of all basement membranes where they regulate an extensive array of tissue functions. Alternative splicing from the laminin α3 gene produces a non‐laminin but netrin‐like protein, Laminin N terminus α31 (LaNt α31). LaNt α31 is widely expressed in intact tissue and is upregulated in epithelial cancers and during wound healing. In vitro functional studies have shown that LaNt α31 can influence numerous aspects of epithelial cell behavior *via* modifying matrix organization, suggesting a new model of laminin auto‐regulation. However, the function of this protein has not been established in vivo. Here, a mouse transgenic line was generated using the ubiquitin C promoter to drive inducible expression of LaNt α31. When expression was induced at embryonic day 15.5, LaNt α31 transgenic animals were not viable at birth, exhibiting localized regions of erythema. Histologically, the most striking defect was widespread evidence of extravascular bleeding across multiple tissues. Additionally, LaNt α31 transgene expressing animals exhibited kidney epithelial detachment, tubular dilation, disruption of the epidermal basal cell layer and of the hair follicle outer root sheath, and ~50% reduction of cell numbers in the liver, associated with depletion of hematopoietic erythrocytic foci. These findings provide the first in vivo evidence that LaNt α31 can influence tissue morphogenesis.

**Abbreviations**
BM: basement membrane
DMEM: Dulbecco's Modified Eagle Medium
ECM: extracellular matrix
hK14: human keratin 14
IP: intraperitoneal injection
LaNt α31: laminin N‐terminus α31
LE: laminin‐type epidermal growth factor‐like domain
LM: laminin
LN: laminin N‐terminal
mEFs: mouse embryonic fibroblasts
SDS‐PAGE: sodium dodecyl sulfate polyacrylamide gel electrophoresis

## 1 INTRODUCTION

Basement membranes (BMs) are specialized extracellular matrix (ECM) structures with essential and diverse roles in regulating cell and tissue behavior; including differentiation, cell adhesion, and migration.^1^, ^2^ BMs not only provide the mechanical attachment points that support sheets of cells to resist stresses but also influence signaling cascades via direct binding to cell surface receptors or indirectly through the sequestration and controlled release of growth factors, or mechanically by providing biomechanical cues, as reviewed in.^3^, ^4^ BMs are also dynamic structures that are remodeled in terms of composition and structure throughout life, with the most extensive changes occurring during development.^5^, ^6^ At the core of every BM are two networks of structural proteins; type IV collagens and laminins (LMs).^7^

Each LM is an obligate αβγ heterotrimer formed from one of five α chains (*LAMA1*‐*5*), one of three β chains (*LAMB1*‐*3*) and one of three γ chains (*LAMC1*‐*3*), with each chain displaying spatio‐temporal distribution patterns, as reviewed in.^8^, ^9^, ^10^, ^11^ Assembly of LM structural networks involves LM‐LM interactions *via* their laminin N‐terminal (LN) domains forming a ternary node comprised of one LN domain from each of the α, β and a γ chains.^12^, ^13^ These αβγ LN ternary nodes assemble in a two‐step process involving an initial rapid formation of unstable βγ LN intermediate which is then stabilized through the incorporation of an α LN domain.^14^, ^15^, ^16^, ^17^ The biological importance of these LN‐LN interactions is exemplified by a group of human disorders where missense mutations affecting the LN domains of the *LAMA2*, *LAMB2* or *LAMA5* genes give rise to syndromic disorders; muscular dystrophy in merosin‐deficient muscular dystrophy for LAMA2, kidney and ocular developmental defects in Pierson syndrome for LAMB2, or defects in kidney, craniofacial and limb development for LAMA5.^18^, ^19^, ^20^, ^21^, ^22^, ^23^ Although these disorders demonstrate that LM network assembly is essential for homeostasis of numerous tissues, not all LM chains contain an LN domain. Specifically, LMα4, which is expressed at high levels in the vasculature, and the LMα3a and LMγ2 chains, which are abundant in surface epithelium including the skin, have shortened amino termini which lack this domain but yet still form functional BMs.^9^, ^24^, ^25^, ^26^, ^27^ This raises questions of whether LN domains are important in all tissue contexts or whether additional proteins compensate for the intrinsic inability of these LMs to form networks.

Alongside their main LM transcripts, the *LAMA3* and *LAMA5* genes produce short transcripts encoding proteins that are unable to trimerize to form LM heterotrimers, but which contain LN domains as their characteristic feature.^24^ At least one of these “laminin N terminus” (LaNt) proteins encodes a functional protein, LaNt α31, comprised of the LMα3 LN domain followed by a short stretch of laminin‐type epidermal growth factor‐like (LE) domains and a unique C‐terminal region with no conserved domain architecture. In addition to the LaNt proteins, the laminin‐superfamily includes the netrin genes which also encode proteins with either a β or γ laminin‐like LN domain, stretches of LE repeats and unique C‐terminal regions (as reviewed in Ref. [28]). Furthermore, proteolytic processing of LMs has also been identified as releasing similar LN domain containing fragments from LMα1,^29^ LMβ1,^30^ and LMα3b.^31^ Some of the LN domain‐containing netrin proteins and cryptic fragments have cell surface receptor binding capabilities and can act as signaling molecules (reviewed in Ref. [32]). However, netrin‐4 also has LM‐network disrupting capabilities due to its unusually high affinity for the LMγ1 LN domain^33^, ^34^ and when netrin‐4 is overexpressed in vivo, it causes increased lymphatic permeability.^35^ The netrin‐4 LN domain has greatest homology with LM β LN domains whereas LaNt α31 contains an exact version of the LMα3b LN domain, which has lower affinity for LN domains; therefore, although LaNt α31 could act similarly to these proteins, it likely plays a different role depending on the LM context.

LaNt α31 is expressed in the basal layer of epithelia in the skin,^24^ cornea^36^ and digestive tract, the ECM around terminal duct lobular units of the breast and alveolar air sacs in the lung, and is widely expressed by endothelial cells.^37^ Increased expression is associated with breast ductal carcinoma and in vitro overexpression leads to a change in the mode of breast cancer cell invasion through LM‐rich matrices.^38^ LaNt α31 is also transiently upregulated during re‐epithelialization of ex vivo corneal burn wounds and in limbal stem cell activation assays.^36^ In epidermal and corneal keratinocytes, knockdown or overexpression experiments revealed that modulating LaNt α31 levels leads to reduced migration rates and changes to cell‐to‐matrix adhesion.^24^, ^39^ Increased expression LaNt α31 also caused changes to LM332, including formation of tight clusters beneath cells and increasing the proteolytic processing of LMα3 by matrix metalloproteinases.^39^

Although the previous in vitro findings all support LaNt α31 as being a mediator of cell behavior, the in vivo impact is as yet unknown, in particular the role it plays in matrixes that are actively being remodeled. Here, we present the first in vivo study of LaNt α31 overexpression in newly developed mouse transgenic models. Analysis revealed that induction of LaNt α31 expression during embryogenesis leads to widespread extravascular red blood cell accumulation associated with capillary BM disruption.

## 2 MATERIALS AND METHODS

### 2.1 Ethics

All procedures were licensed by the UK Home Office under the Animal (Specific Procedures) Act 1986, project license numbers (PPL) 70/9047 and 70/7288. All mice were housed and maintained within the University of Liverpool Biological Services Unit in specific pathogen‐free conditions in accordance with UK Home Office guidelines. Food and water were available ad libitum.

### 2.2 Antibodies

Rabbit monoclonal antibodies against the influenza hemagglutinin epitope (HA) (C29F4, Cell Signalling Technology) were used for immunoblotting at 67 ng/ml. Goat polyclonal antibodies against DDDDK (equivalent to Flag sequence, ab1257, Abcam), rabbit polyclonal antibodies against 6X‐His (ab137839, Abcam), and rabbit polyclonal antibodies against lamin A/C (4C11, Cell Signalling Technology) were used at 1 µg/ml for immunoblotting. Mouse monoclonal antibodies against LaNt α31^36^ were used at 0.225 µg/ml for immunoblotting. Rabbit polyclonal antibodies against mCherry (ab183628, Abcam) were used at 2.5 µg/ml for immunofluorescence. Antibodies against laminin α4‐subunit (clone 377b) and laminin α5‐subunit (clone 504) were kindly provided by Prof. L. Sorokin (Institute of Physiological Chemistry and Pathobiochemistry; Münster University).^40^ J18 polyclonal antiserum was raised in a rabbit using rat LM 332 purified from ECM preparations of 804G cells, as previously described.^41^ Alexa fluor 647 conjugated goat anti‐rabbit IgG recombinant secondary antibodies were obtained from Thermo Fisher Scientific and used at 2 µg/ml for indirect immunofluorescence microscopy.

### 2.3 pUbC‐LoxP‐LaNtα31‐T2A‐tdTomato

A gBlock was synthesized (Integrated DNA Technologies) containing *Nde*I and *Nhe*I restriction enzyme sites, T7 promoter binding site,^42^ Kozak consensus sequence,^43^ Igκ secretion signal (METDTLLLWVLLLWVPGSTGD),^44^ LaNt α31‐encoding cDNA (amino acids 38–488),^24^ Flag (DYKDDDDK)^45^ and HA (YPYDVPDYA)^46^ tag sequences, T2A sequence (EGRGSLLTCGDVEENPGP),^47^ and *BamH*I. The gBlock DNA was inserted into pCSCMV:tdTomato (a gift from Gerhart Ryffel, Addgene plasmid #30530; http://n2t.net/addgene:30530; RRID:Addgene_30530) using *Nde*I and *BamH*I (New England Biolabs), to produce pCS‐LaNtα31‐T2A‐tdTomato. LaNtα31‐T2A‐tdTomato was then removed from this backbone using *Nhe*I and *EcoR*I, and inserted into a vector containing the Ubiquitin C (UbC) promoter and a floxed stop cassette, all flanked by cHS4 insulator elements, producing pUbC‐LoxP‐LaNtα31‐T2A‐tdTomato.

### 2.4 Cloning procedures

Restriction digests were set up with 1 μg of plasmid DNA, 1 µg of PCR product, or 100 ng of gBlock DNA, 20 U of each enzyme and CutSmart buffer (50 mM potassium acetate, 20 mM Tris‐acetate, 10 mM magnesium acetate, 100 μg/ml BSA (New England Biolabs) and incubated at 37°C for 1 h. Enzymatic activity was inactivated by 20 min incubation at 65°C. PCR or cloning products were separated using 1% (w/v) agarose gels (Thermo Fisher Scientific) dissolved in 1 × TAE electrophoresis buffer (40 mM Tris pH 7.6, 20 mM acetic acid, 1 mM EDTA) containing ethidium bromide (Sigma Aldrich), and visualized using a UV transilluminator ChemiDoc MP System (BioRad). DNA bands were excised from the gel and purified using the GenElute^™^ Gel Extraction Kit, following manufacturer's protocol (Sigma Aldrich). Purified inserts were ligated into vectors at 3:1 molar ratios, either using Instant Sticky‐end Ligase Master Mix (New England Biolabs) following manufacturers protocol, or using 400 U of T4 DNA ligase and 1× reaction buffer (50 mM Tris‐HCl, 10 mM MgCl_2_ 1 mM ATP, 10 mM DTT, New England Biolabs) at 16°C overnight, followed by enzymatic inactivation at 65°C for 10 min. Ligated DNA was heat‐shock transformed into One‐Shot TOP10 chemically competent E. coli cells (Thermo Fisher Scientific) following manufacturer's protocol, then plated onto LB plates containing the appropriate antibiotic (100 μg/ml ampicillin, 50 μg/ml kanamycin or 25 μg/ml chloramphenicol, Sigma Aldrich). Plasmid DNA was extracted from bacteria using the GenElute^™^ Plasmid Miniprep Kit (Sigma Aldrich), following the manufacturer's protocol. Plasmids were sequenced by DNASeq (University of Dundee).

### 2.5 Cell culture

KERA‐308 murine epidermal keratinocyte cells,^48^ were purchased from CLS (Cell Lines Service GmbH) and maintained in high glucose (4.5 g/L) Dulbecco's Modified Eagle Medium (DMEM, Sigma Aldrich) supplemented with 10% fetal calf serum (FCS, LabTech) and 2 mM L‐glutamine (Sigma Aldrich). HEK293A cells were maintained in DMEM supplemented with 10% FCS and 4 mM L‐glutamine.

### 2.6 Cell transfections

1 × 10^6^ KERA‐308 or 4 × 10^5^ HEK293A cells were seeded in 6‐well plates (Greiner‐BioOne) 24 h prior to transfection. For KERA‐308 cells, 2 µg of hK14‐LaNtα31‐T2A‐mCherry or LaNt‐α31‐pSec‐Tag and 2 µl Lipofectamine 2000 (Thermo Fisher Scientific) were used. For HEK293A cells, either 1 µg pCAG‐Cre:GFP and 2 µl Lipofectamine 2000, 2 µg of pUbC‐LoxP‐LaNtα31‐T2A‐tdTomato and 5 µl Lipofectamine 2000, or 2 µg of pUbC‐LoxP‐LaNtα31‐T2A‐tdTomato, 1 µg of pCAG‐Cre:GFP and 7 µl Lipofectamine 2000 (Thermo Fisher Scientific), were mixed with 2 ml of Gibco^™^ Opti‐MEM^™^ Reduced Serum Medium (Thermo Fisher Scientific) and incubated for 10 min at room temperature. The DNA‐lipofectamine complex was added to the wells, and the media was replaced with DMEM high glucose after 6 h.

### 2.7 Explant culture method

Hair was removed from mouse skin tissue using Veet hair removal cream (Reckitt Benckiser) and the skin washed in Dulbecco's phosphate buffered saline (DPBS) containing 200 U/ml penicillin, 200 U/ml streptomycin, and 5 U/ml amphotericin B1 (all Sigma Aldrich). The skin was then dissected into 2–3 mm^2^ pieces using a surgical scalpel and 3 or 4 pieces placed per well of a 6‐well dish (Greiner Bio‐One, Kremsmünster, Austria) with the dermis in contact with the dish. 300 µl of DMEM supplemented with 20% FCS, 2 mM L‐glutamine, 200 µg/ml penicillin, 200 µg/ml streptomycin, and 5 µg/ml fungizone (all Sigma Aldrich) was added to the wells. After 24 h, each well was topped up with 1 ml of media, and the media was replenished every 48 h thereafter.

### 2.8 Transgenic line establishment

Generation of transgenic mice were carried out based on the protocol described in Ref. [49] C57Bl6CBAF1 females (Charles River Laboratories) between 6 and 8 weeks were superovulated by intraperitoneal (IP) injections of 5 IU pregnant mare's serum gonadotrophin (PMSG; in 100 µl H_2_O) (Sigma Aldrich), followed 46 h later by 5 IU of human chorionic gonadotropin (hCG, Sigma Aldrich). Treated females were mated with C57Bl6CBAF1 males overnight. Mated females were identified from the presence of copulation plugs, anaesthetized, and oviducts removed and dissected in M2 media (Millipore). Day‐1 oocytes (C57BL/6Jx CBA F1) were transferred into clean media by mouth pipetting. Cumulus cells were removed by hyaluronidase (300 µg/ml, Merck) treatment in M2 media (Millipore, Speciality Media, EmbryoMax) with gentle shaking until detached from the egg surface. Oocytes were then rinsed and transferred to M16 media (Millipore, Speciality Media, EmbryoMax) ready for injection.

DNA was diluted to a final concentration of 2 ng/µl in embryo water (Sigma Aldrich) and filter‐purified using Durapore‐PVDF 0.22 µM centrifuge filters (Merck). Injection pipettes were used to pierce the outer layers of the oocyte and to inject DNA. DNA was injected into the pronuclei of the oocyte. Undamaged eggs were transferred to clean M16 media and incubated at 37°C until transferred into pseudopregnant CD1 females on the same day. Meanwhile, pseudopregnant females were obtained by mating vasectomized CD1 males overnight. Copulation plugs were checked and females were used 1 d post‐coitum. Females were anaesthetized by inhalation of isoflurane (Sigma Aldrich). Thirty injected oocytes were transferred to plugged pseudopregnant female oviducts through the infundibulum.

In generating the pUbC‐LoxP‐LaNtα31‐T2A‐tdTomato line, 460 mouse zygotes were injected over four sessions. 87% of these zygotes survived and were transferred into 11 recipient CD1 mothers. From these mothers, 42 pups were born. Of the 10 F0 mice that gave a positive genotype result, four passed on the transgene to the F1 generation. Mice that did not pass on the transgene to the F1 generation were culled, the four F0 mice were mated and one line was continued for investigation.

R26CreERT2 (Jax Lab 008463)^50^ mice were purchased from The Jackson Laboratory.

### 2.9 In vivo transgene induction

Tamoxifen (Sigma Aldrich) was dissolved in corn oil (Sigma Aldrich) and administered IP at 25 or 75 mg/kg. Progesterone (Sigma Aldrich) was dissolved in corn oil (Sigma Aldrich) and was co‐administered alongside tamoxifen at half of the corresponding tamoxifen dose (12.5 or 25 mg/kg).

### 2.10 DNA extraction

Four weeks after birth, ear notches were collected from mouse pups and digested in 100 µl lysis buffer (50 mM Tris‐HCl pH 8.0, 0.1 M NaCl, 1% SDS, 20 mM EDTA) and 10 µl of proteinase K (10 mg/ml, all Sigma Aldrich) overnight at 55°C. The following day, samples were cooled, spun at 13 000 rpm for 3 min and the supernatant transferred to clean 1.5 ml tubes (Eppendorf). An equal volume of isopropanol (Sigma Aldrich) was added, gently inverted and spun at 13 214 g, and supernatant discarded. Pellets were washed with 500 µl of 70% EtOH (Sigma Aldrich), then air‐dried for 10 min, and resuspended in 50 µl ddH_2_O.

### 2.11 PCR

50 ng of genomic DNA was mixed with 12.5 µl of REDtaq ReadyMix PCR Reaction Mix (20 mM Tris‐HCl pH 8.3, 100 mM KCl, 3 mM MgCl_2_, 0.002% gelatin, 0.4 mM dNTP mix, 0.06 unit/ml of Taq DNA Polymerase, Sigma Aldrich) and 0.5 µM of each primer; ddH_2_O was added to make the reaction mixture up to 25 µl. Primer pairs for genotyping were as follows: LaNt α31 to tdTomato Forward 5′‐ATCTATGCTGGTGGAGGGGT‐3′, Reverse 5′‐TCTTTGATGACCTCCTCGCC‐3′; Cre Forward 5′‐GCATTACCGGTCGATGCAACGAGTGATGAG‐3′, Reverse 5′‐GAGTGAACGAACCTGGTCGAAATCAGTGCG‐3′; Recombination Forward 5′‐TCCGCTAAATTCTGGCCGTT‐3′, Reverse 5′‐GTGCTTTCCTGGGGTCTTCA‐3′ (all from Integrated DNA Technologies). Cycle conditions were as follows: Genotyping – 1 cycle of 95°C for 5 min, 35 cycles of 95°C for 15 s; 56°C for 30 s; 72°C for 40 s, followed by a final cycle of 72°C for 5 min. For assessing recombination: 1 cycle of 95°C for 5 min, 35 cycles of 95°C for 15 s; 60°C for 30 s; 72°C for 90 s, followed by a final cycle of 72°C for 7 min. PCR products were separated by gel electrophoresis and imaged using a BioRad Gel Doc XR+ System.

### 2.12 Sodium dodecyl sulfate polyacrylamide gel electrophoresis (SDS‐PAGE) and western immunoblotting

Cells were homogenized by scraping into 90 µl Urea/SDS buffer (10 mM Tris‐HCl pH 6.8, 6.7 M urea, 1% w/v SDS, 10% v/v glycerol and 7.4 µM bromophenol blue, containing 50 µM phenylmethysulfonyl fluoride and 50 µM N‐methylmaleimide, all Sigma Aldrich). Lysates were sonicated and 10% v/v β‐mercaptoethanol (Sigma Aldrich) added. Proteins were separated by SDS‐PAGE using 10% polyacrylamide gels; 1.5 M Tris, 0.4% w/v SDS, 10% acrylamide/bis‐acrylamide (all Sigma Aldrich), electrophoresis buffer; 25 mM tris‐HCl, 190 mM glycine, 0.1% w/v SDS, pH 8.5 (all Sigma Aldrich). Proteins were transferred to a nitrocellulose membrane using the TurboBlot^™^ system (BioRad) and blocked at room temperature in Odyssey® TBS‐Blocking Buffer (Li‐Cor BioSciences) for 1 h. The membranes were probed overnight at 4°C diluted in blocking buffer, washed 3 × 5 min in PBS with 0.1% Tween (both Sigma Aldrich) and probed for 1 h at room temperature in the dark with IRDye® conjugated secondary Abs against goat IgG (800 CW) and rabbit IgG (680 CW), raised in goat or donkey (LiCor BioSciences), diluted in Odyssey® TBS‐Blocking Buffer at 0.05 µg/ml. Membranes were then washed for 3 × 5 min in PBS with 0.1% Tween, rinsed with ddH_2_O and imaged using the Odyssey® CLX 9120 infrared imaging system (LiCor BioSciences). Image Studio Light v.5.2 was used to process scanned membranes.

### 2.13 Tissue processing

For cryosections, P0 pups were culled by cervical dislocation, and fixed in 4% paraformaldehyde (Merck) for 2 h at 4°C. Samples were cryoprotected in 30% sucrose/PBS solutions then in 30% sucrose/PBS:O.C.T (1:1) solutions (Tissue‐Tek, Sakura Finetek Europe), each overnight at 4°C. Samples were embedded in OCT compound (Tissue‐Tek) and transferred on dry ice. Embedded samples were sectioned at 10 μm using a Leica CM1850 cryostat (Leica). For paraffin sections, Tissues were fixed in 10% neutral buffered formalin (Leica,) for 24 h, then processed through graded ethanol and xylene before being embedded in paraffin wax. 5 μm sections were cut using a rotary microtome RM2235 (Leica), adhered to microscope slides, then dried overnight at 37°C. Sections were dewaxed and rehydrated with xylene followed by a series of decreasing ethanol concentrations.

### 2.14 Hematoxylin and eosin (H&E) staining

Sections were dewaxed and rehydrated with xylene followed by a series of decreasing ethanol concentrations. Sections were then stained in Harris hematoxylin solution (Leica) for 5 min, H_2_O for 1 min, acid alcohol (Leica) for 5 s, H_2_O for 5 min, aqueous eosin (Leica) for 3 min, H_2_O for 15 s, followed by dehydration through graded ethanol and xylene. Slides were coverslipped with DPX mounting media (Sigma Aldrich).

### 2.15 Immunohistochemistry

Slides were incubated in ice‐cold acetone for 10 min, PBS for 10 min, then blocked in PBS containing 10% normal goat serum (NGS) at room temperature for 1 h. Samples were probed with the primary antibodies diluted in PBS‐Tween (0.05%) with 5% NGS at 4°C overnight, washed for 3 × 5 min in PBS‐Tween (0.05%), then probed with secondary antibodies diluted in PBS‐Tween (0.05%) with 5% NGS at room temperature for 1 h. Samples were washed for 3 × 5 min in PBS‐Tween (0.05%), then mounted with VECTASHIELD® Antifade Mounting Medium with DAPI (VECTASHIELD®).

### 2.16 Image acquisition

H&E images were acquired using a Zeiss Axio Scan.Z1 equipped with an Axiocam colour CCD camera using the ZEN Blue software (all from Zeiss). Live cell images were acquired using a Nikon Eclipse Ti‐E microscope (Nikon). Immunofluorescence images of tissues were acquired using a Zeiss LSM 800 confocal microscope (Zeiss).

### 2.17 Transmission electron microscopy

Kidneys and backskin were dissected from the p0 mice and placed immediately into 4% (w/v) paraformaldehyde, 2.5% (w/v) glutaraldehyde in cacodylate pH7.4 for 30 min at room temperature. The samples were then dissected into 3 mm^3^ pieces and placed into fresh fixative and rotated overnight at room temperature. Samples were washed 4 × 5 min with 0.1 M cacodylate buffer, before staining with reduced osmium (final concentration 1% (w/v) OsO_4_, 1.5% (w/v) potassium ferrocyanide, 0.1 M cacodylate buffer) in a Pelco Biowave® Pro (Ted Pella Inc.). Following this, samples were washed 5 × 5 min in ddH_2_O, and incubated overnight in aqueous 1% uranyl acetate at 4°C. After further ddH_2_O washes Samples were dehydrated through increasing concentrations of acetone (30%, 50%, 70%, 90% for 15 min each then 3 × 100%) Samples were infiltrated in 1:1 acetone TAAB 812 medium resin for 2 days followed by 4 × 100% resin for 1 h each, before final embedding and curing at 60°C for 48 h. Tissue was sectioned at 70–75 nm on a ultramicrotome (Leica) and the viewed in a FEI 120 Kv Tecnai Spirtit BioTwin TEM (FEI Company), fitted with a Gatan RIO16 digital camera (Gatan).

### 2.18 Image analysis

Images were processed using either Zen 2.6 (blue edition) (Zeiss) or Fiji/ImageJ (National Institutes of Health).^51^ Stardist plugin^52^ was used for segmentation of nuclei from H&E images. Images were thresholded manually to remove areas containing no tissue in the images. Hemidesmosome number per μm in transmission electron micrographs was determined using the freehand selection tool on Fiji/ImageJ to measure BM length per image and manually counting hemidesmosomes. Hemidesmosome size was determined using the freehand selection tool to measure the length of electron dense plaques at the plasma membrane.

## 3 RESULTS

### 3.1 Inducible LaNt α31 construct validation

**FIGURE 1:**
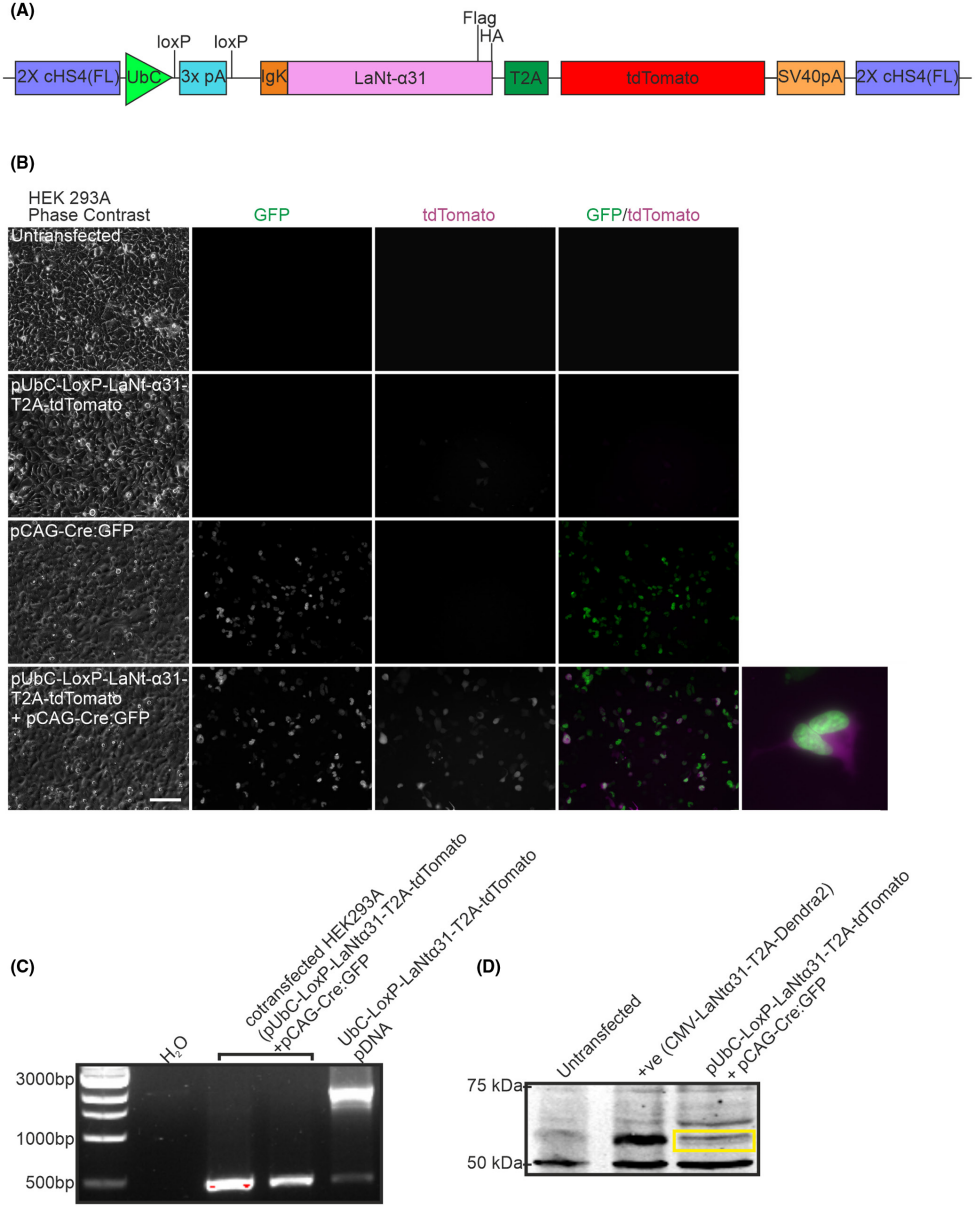
Validation of UbCLaNt Cre‐inducible construct in vitro. (A) Diagram of the pUbC‐LoxP‐LaNt‐α31‐T2A‐tdTomato construct. (B) HEK 293A cells were transfected with pUbC‐LoxP‐LaNt‐α31‐T2A‐tdTomato, pCAG‐Cre:GFP, or pUbC‐LoxP‐LaNt‐α31‐T2A‐tdTomato and pCAG‐Cre:GFP and imaged 48 h after transfection. Scale bar 100 µm. (C) PCR products using primers flanking the stop cassette on DNA extracted from HEK293A cells co‐transfected with pUbC‐LoxP‐LaNt‐α31‐T2A‐tdTomato and pCAG‐Cre:GFP. (D) Western blot of lysates from HEK293 cells either untransfected or transfected with CMV‐ LaNt‐α31‐T2A‐Dendra2 (positive control), or pUbC‐LoxP‐LaNt‐α31‐T2A‐tdTomato and pCAG‐Cre:GFP then probed with anti‐Flag antibodies

To investigate the consequences of LaNt α31 overexpression in vivo, an inducible system for conditional LaNt α31 transgene expression was generated (Figure 1A). An expression construct was created containing the ubiquitin C promoter driving expression of the human LaNt α31 cDNA. To focus our studies on extracellular role of LaNt a31, the native secretion signal was replaced by mouse immunoglobulin κ leader sequence.^44^ This signal sequence has been used to increase protein secretion efficiency in mammalian cells.^53^, ^54^, ^55^ Flag and HA epitope tags were added to the C‐terminus of the LaNt α31 coding region. A T2A element was included to enable expression of tdTomato from the same transgene but not directly fused to LaNt α31.^47^ A floxed stop‐cassette was inserted between the promoter and the start of the construct to prevent transgene expression until Cre‐mediated removal of this cassette. The entire construct was flanked with the cHS4 β‐globin insulator to protect against chromatin‐mediated gene silencing^56^ (Figure 1A). Restriction enzyme digests and plasmid sequencing confirmed the assembled pUbC‐LoxP‐LaNtα31‐T2A‐tdTomato plasmid.

To confirm the construct expressed only following exposure to Cre recombinase, the pUbC‐LoxP‐LaNtα31‐T2A‐tdTomato was co‐transfected alongside pCAG‐Cre:GFP, encoding GFP‐tagged Cre recombinase, into HEK293A cells. tdTomato signal was observed only in cells transfected with both plasmids (Figure 1B). PCR using primers flanking the STOP cassette also confirmed that the cassette was removed only in cells transfected with both plasmids (Figure 1C). Western blotting using polyclonal anti‐Flag antibodies confirmed expression of the predicted ~57 kDa band in co‐transfected cell lysates (Figure 1D), this also confirmed that the T2A element was cleaved in the final product releasing the tdTomato tag. Together, these results demonstrated that the pUbC‐LoxP‐LaNtα31‐T2A‐tdTomato plasmid allows for the Cre‐inducible expression of LaNt α31 and tdTomato.

### 3.2 Generation and validation of a LaNt α31 transgenic mouse line

The pUbC‐LoxP‐LaNtα31‐T2A‐tdTomato construct was linearized, and transgenic F0 mice generated by pronuclear microinjection into oocytes. To confirm transgene expression, F0 mice were mated with WT (C57BL/6J) mice, embryos were collected at E11.5, and mouse embryonic fibroblasts (mEFs) were isolated from the embryos. Presence of the UbC‐LoxP‐LaNtα31‐T2A‐tdTomato transgene (hereafter UbCLaNt) was confirmed by PCR (Figure S1A). mEFs were transduced with an adenovirus encoding codon‐optimized Cre recombinase (ad‐CMV‐iCre). Analysis by immunoblotting with anti‐HA‐antibodies (Figure S1B) revealed a ~57 KDa band and fluorescence microscopy confirmed tdTomato expression in samples containing both the UbC‐LaNt transgene and the ad‐CMV‐iCre only (Figure S1C).

**FIGURE 2:**
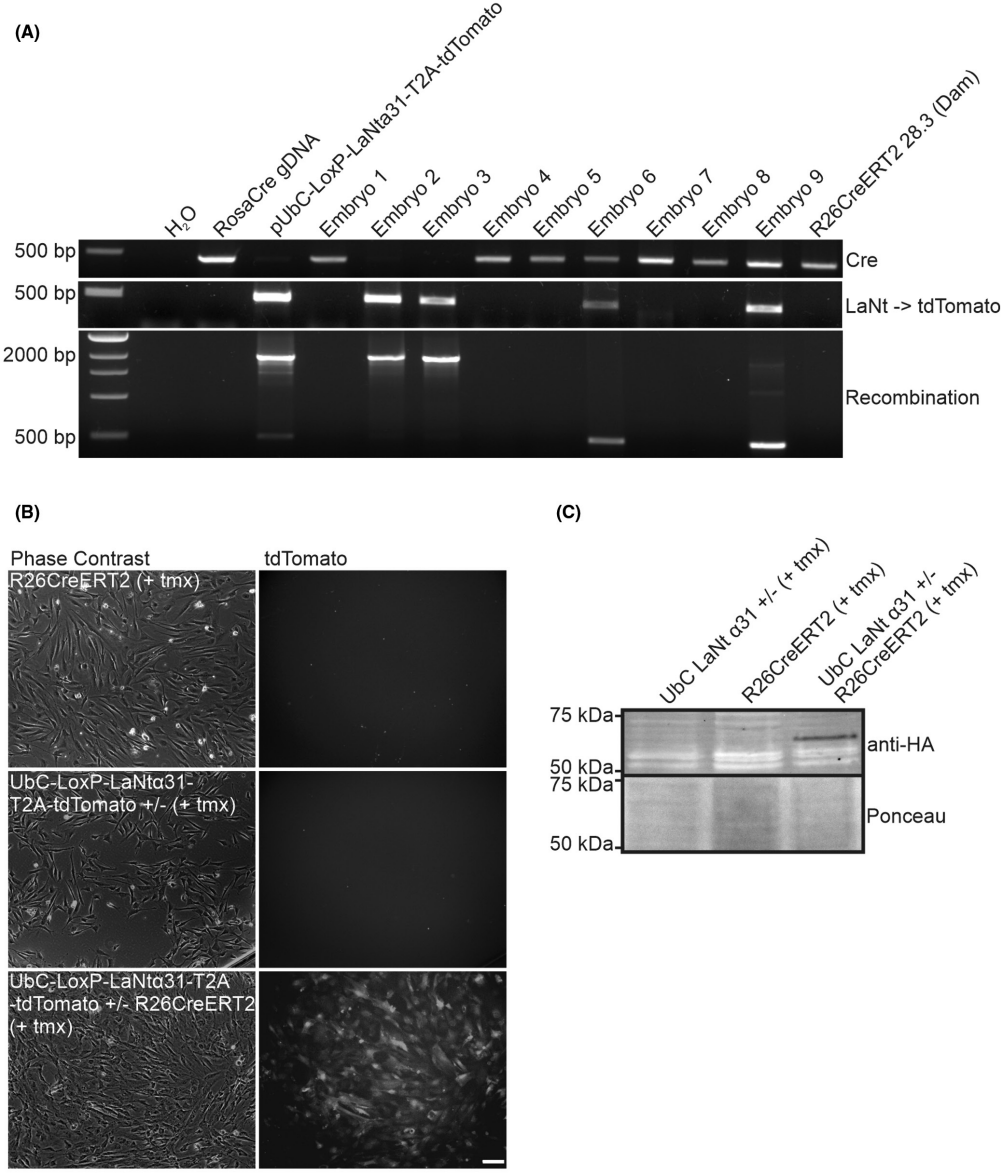
UbCLaNtα31 x R26CreERT2 ER transgenic mice express the UbC‐LaNtα31 transgene following exposure to tamoxifen. (A) PCR products on DNA extracted from transgenic mouse from UbCLaNtα31 x R26CreERT2 mating embryos using primers flanking the stop cassette. (B) Phase contrast and fluorescence microscopy images of explanted cells from UbCLaNtα31::R26CreERT2 embryos. Scale bar =100 µm. (C) Western blot of lysates from UbCLaNtα31::R26CreERT2 embryo explants processed with anti‐HA antibodies

Male UbCLaNt mice were mated with females from the tamoxifen‐inducible ubiquitous Cre line R26CreERT2. Transgene expression was induced by IP injection of tamoxifen at E13.5, and embryos collected at E19.5. PCR confirmed that Cre/LoxP mediated recombination only occurred in the embryos with both the UbCLaNt and the R26CreERT2 transgenes (Figure 2A). Explants were generated from the skin of these embryos, and only the explants grown from double transgenic embryos exhibited tdTomato expression by fluorescence microscopy (Figure 2B) and HA‐tagged LaNt α31 expression by western immunoblotting (Figure 2C). Together, these data confirmed the generation of tamoxifen‐inducible LaNt α31 mouse line, without detectable leakiness (UbCLaNt::R26CreERT2).

### 3.3 UbCLaNt::R26CreERT2 expression in utero causes death and localized regions of erythema at birth

**FIGURE 3:**
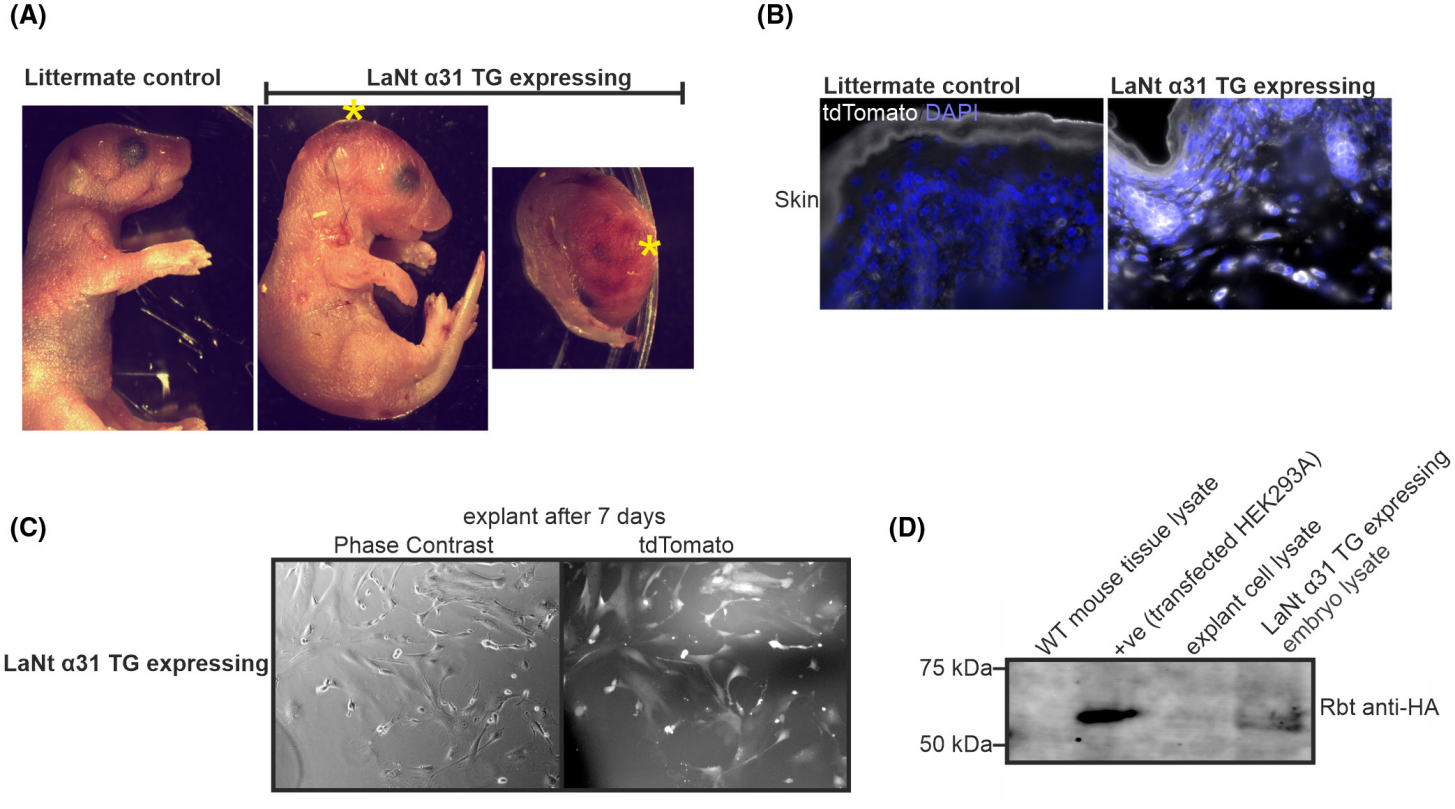
Transgenic mice overexpressing LaNtα31 display localized regions of erythema. (A) Representative images of UbCLaNtα31::R26CreERT2 embryos. Animals subsequently confirmed as expressing the LaNt α31 transgene are labeled as LaNt α31 TG expressing. *Indicates regions of visible erythema. (B) Representative fluorescence microscopy UbCLaNtα31::R26CreERT2 OCT sections tdTomato fluorescence. Scale bar =100 µm. (C) Fluorescence microscopy images of explanted cells from LaNt α31 TG expressing. Scale bar =100 µm. (D) Western blot of tissue lysates from WT, UbCLaNtα31::R26CreERT2 embryos or explanted cells processed with anti‐HA antibodies. HEK293A cells cotransfected with the LaNt α31 transgene expression construct and Cre‐GFP expression construct are included as a positive control

To determine the impact of LaNt α31 during development, tamoxifen was administered to pregnant UbCLaNt::R26CreERT2 mice at E15.5 via gavage and pregnancies allowed to continue to term. Across three litters from three different mothers, two from six pups, three from five pups, and one from five pups respectively were intact but not viable at birth, while the remaining littermates were healthy. The non‐viable pups displayed localized regions of erythema with varying severity between the mice, but were otherwise fully developed and the same size as littermates (Figure 3A). Endpoint PCR genotyping using primers amplifying the LaNt α31 transgene and Cre recombinase transgene confirmed mice possessed both transgenes (Figure S2A). To confirm that the lack of viability was associated with transgene expression, OCT‐embedded skin sections of UbCLaNt::R26CreERT2 were imaged using confocal microscopy, revealing tdTomato fluorescence only in the non‐viable animals (Figure 3B) and skin explants were established and tdTomato fluorescence in explants from non‐viable pups was confirmed by microscopy (Figure 3C). Western immunoblot analysis of total protein extracts from the explanted cells and from whole embryo lysates also revealed transgene expression in non‐viable pups, although expression levels varied between the mice (Figure 3D). Together these data confirmed that only non‐viable mice expressed the LaNt α31 transgene. Hereafter, UbCLaNt::R26CreERT2 animals are therefore labeled as either “LaNt α31 TG‐expressing” or, for non‐expressing, “littermate controls”.

To identify LaNt α31 effects at the tissue level, the pups were formalin‐fixed and paraffin‐embedded then processed for H&E staining and immunohistochemistry. All organs were present in the mice and appeared intact at the macroscopic level. A consistent feature in every transgenic animal was extensive evidence of bleeding within tissues. Indeed, although there was mouse‐to‐mouse variability in extent of this bleeding, every major organ in all animals were affected to some extent.

We focused our attention on kidney, skin and lung as examples of tissues where the BMs with distinct differences in LM composition and where LaNt α31 could elicit context‐specific effects. Each of these three tissues also express LaNt α31 in adult human tissue, and are, therefore, tissues where dysregulation of expression regulation could be physiologically relevant.^37^ Specifically, the predominant LMs in the kidney contain three LN domains, and mutations affecting LM polymerization lead to Pierson syndrome,^19^, ^57^, ^58^, ^59^, ^60^ whereas the major LM in the skin contains one LN domain, LM332, and loss of function leads to skin fragility, reviewed in,^61^ and granulation tissue disorders.^62^, ^63^ In the lung, LM311, a two LN domain LM, is enriched^64^, ^65^ and absence of LM α3 is associated with pulmonary fibrosis.^66^

### 3.4 LaNt α31 overexpression leads to epithelial detachment, tubular dilation and interstitial bleeding in the kidney and disruption of capillary BM integrity

**FIGURE 4:**
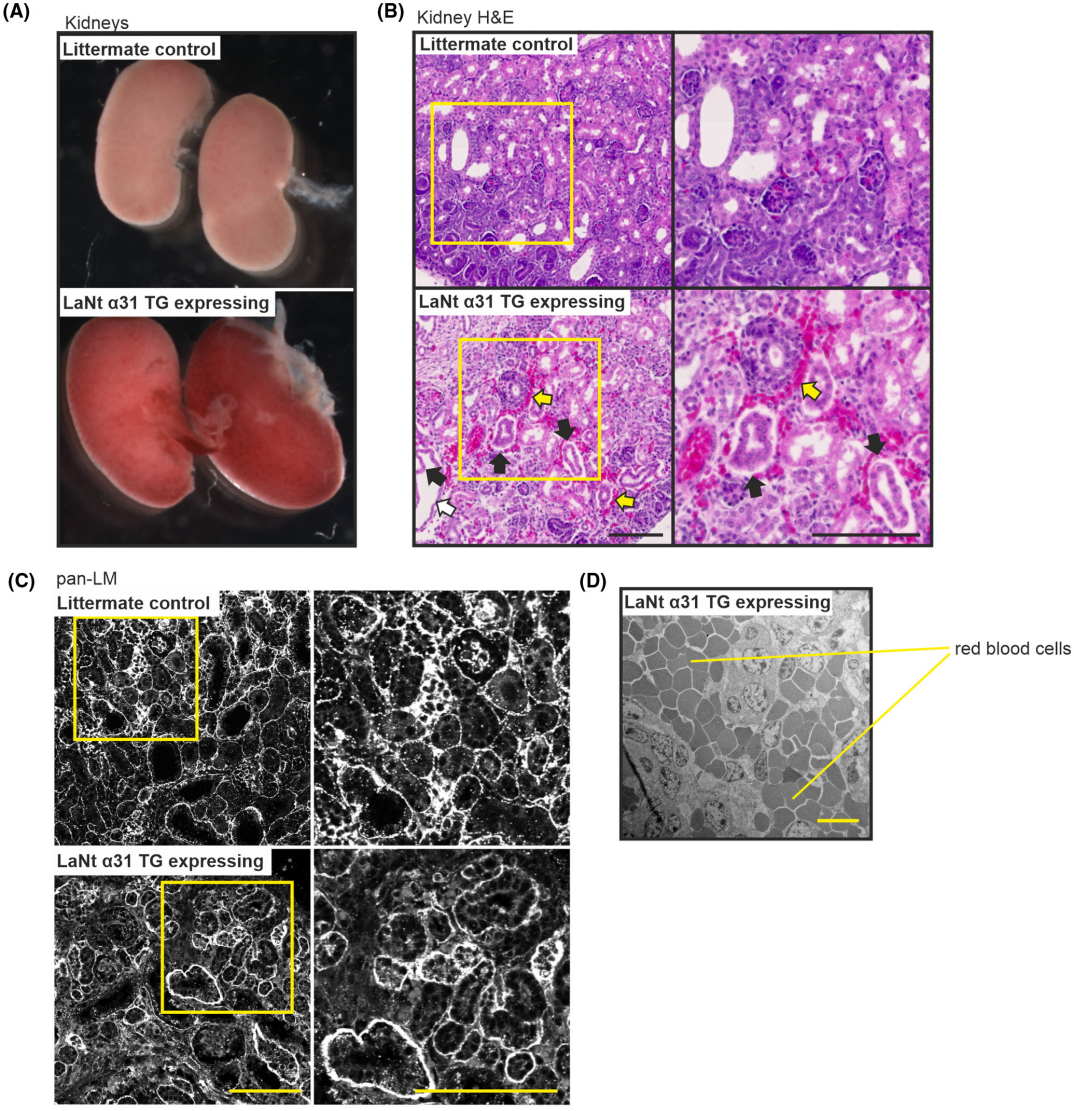
LaNt α31 overexpression leads to epithelial detachment, tubular dilation and interstitial bleeding in the kidney and disruption of capillary basement membrane integrity. (A) Representative images of whole kidneys of newborn UbCLaNtα31::R26CreERT2 mouse kidneys from non‐expressing littermate controls (top) or LaNt α31 TG expressing animals (bottom). (B) Representative images of H&E stained FFPE sections (5 μm) of newborn littermate controls of LaNt α31 TG expressing mouse kidneys. Right column shows areas of increased magnification. Black arrows point to areas of epithelial detachment. White arrows point to tubular dilation. Yellow arrows point to areas of interstitial bleeding. (C) FFPE sections (5 μm) from littermate controls or LaNt α31 TG expressing animals processed for immunohistochemistry with pan‐laminin polyclonal antibodies. Right column shows areas of increased magnification. Scale bars =100 μm

Dissected kidneys from the transgene‐expressing animals were markedly darker than non‐expressing animals (Figure 4A). Histological examination confirmed that this difference reflected differences in the vessels of the kidney, with extensive bleeding into the interstitial and sub tubular surroundings (Figure 4B, yellow arrows). Detachment of the lining epithelia in collecting ducts and uteric bud segments was also apparent (Figure 4B, black arrows). Indirect IF processing of tissue using pan‐LM antibodies revealed LM localization to be largely unchanged (Figure 4C). However, ultrastructural examination by transmission electron microscopy identified that the majority of extravascular red blood cells present in the tissue were outside of capillary structures (Figure 4D).

### 3.5 LaNt α31 overexpression disrupts epidermal basal cell layer organization

**FIGURE 5:**
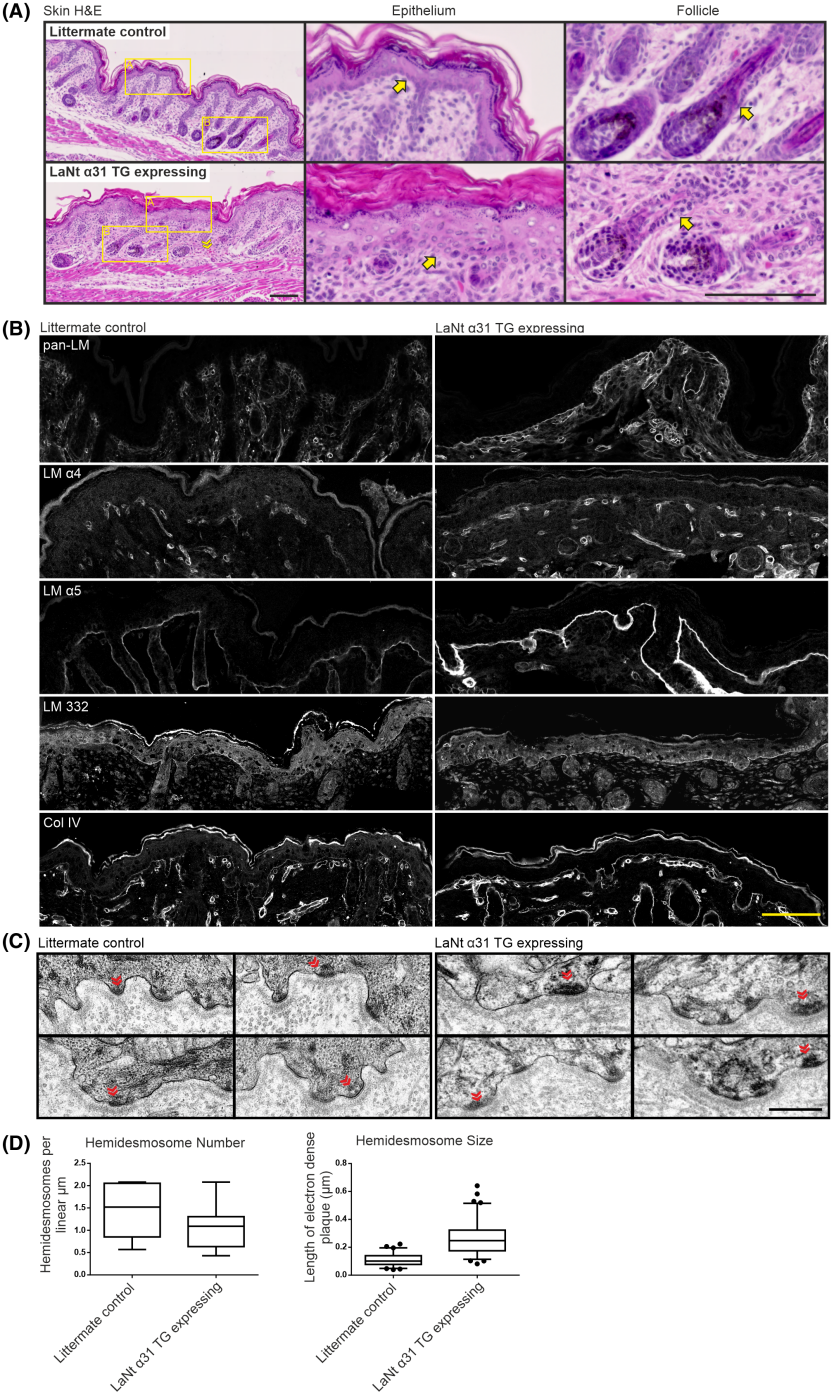
LaNt α31 overexpression disrupts epidermal‐dermal cell organization. (A) H&E staining of FFPE sections (5 μm) of newborn UbCLaNtα31::R26CreERT2 transgenic mice dorsal skin. Upper panel non‐expressing littermate controls, lower panels LaNt α31TG expressing animals. Yellow chevrons indicate areas of extravascular erythrocytes. Middle and right columns show increased magnification of the epithelium or hair follicles respectively. Yellow arrows indicate basal layer of epithelial cells. Scale bar =100 μm. (B) Littermate controls (left) or LaNt α31 TG expressing OCT sections (10 μm) processed for immunohistochemistry with anti‐laminin 111 (pan‐LM), anti‐laminin α4 (LM α4), anti‐laminin α5 (LM α5), anti‐laminin 332 (LM 332) and anti‐Type IV collagen (Col IV). Scale bar =50 μm. (C) Transmission electron micrographs of littermate control or LaNt α31 TG expressing skin sections imaged at the dermal‐epidermal junctions. Chevrons indicate hemidesmosomes. Scale bar =0.5 µm. (D) Box and whisker graphs of quantification of hemidesmosome number per µm of basement membrane (*n* = 12 and 17 images), and of size of the hemidesmome measured as the length of the electron dense plaque at the cell membrane (*n* = 67 and 81 hemidesmosomes). Boxes represent 25th–75th percentile with line at median, whiskers 5th and 95th percentile. Dots represent outliers

Histological examination of the dorsal skin of the LaNt α31 TG expressing mice revealed localized disruption of the epidermal basal cell layer, with a loss of the tight cuboidal structure of the stratum basale (Figure 5A). Basal layer disruption was also observed in the outer root sheath of the hair follicles (Figure 5A). There was no evidence of blistering at the dermal‐epidermal junction. However, extravascular erythrocytes were observed through the skin (Figure 5A, yellow chevron). Indirect IF processing revealed that the localization of LMα5, LM 332, and type IV collagen was unchanged in LaNt α31 TG expressing animals, although increased immunoreactivity of LM α5 was observed (Figure 5B, Figure S3). This increase in immunoreactivity was also observed in samples processed with a pan‐LM antibody (Figure 5B). The immunoreactivity of LMα4 appeared unchanged in vessels; but this laminin chain was also detected at the dermal‐epidermal junction in LaNt α31 TG expressing animals (Figure 5B). Ultrastructural analyses of the dermal‐epidermal junction revealed no major disruption to the BM. However, in the LaNt α31 TG specimens, hemidesmosomes were larger (Figure 5C, chevrons, Figure 5D, littermate median 0.11 95% CI 0.09–0.12 µm, LaNt α31 TG median 0.26 95% CI 0.24–0.28 µm, *p *<.001 Mann‐Whitney test).

### 3.6 Mice expressing the LaNt α31 transgene display structural differences in the lung and a reduction of hematopoietic colonies in the liver

**FIGURE 6:**
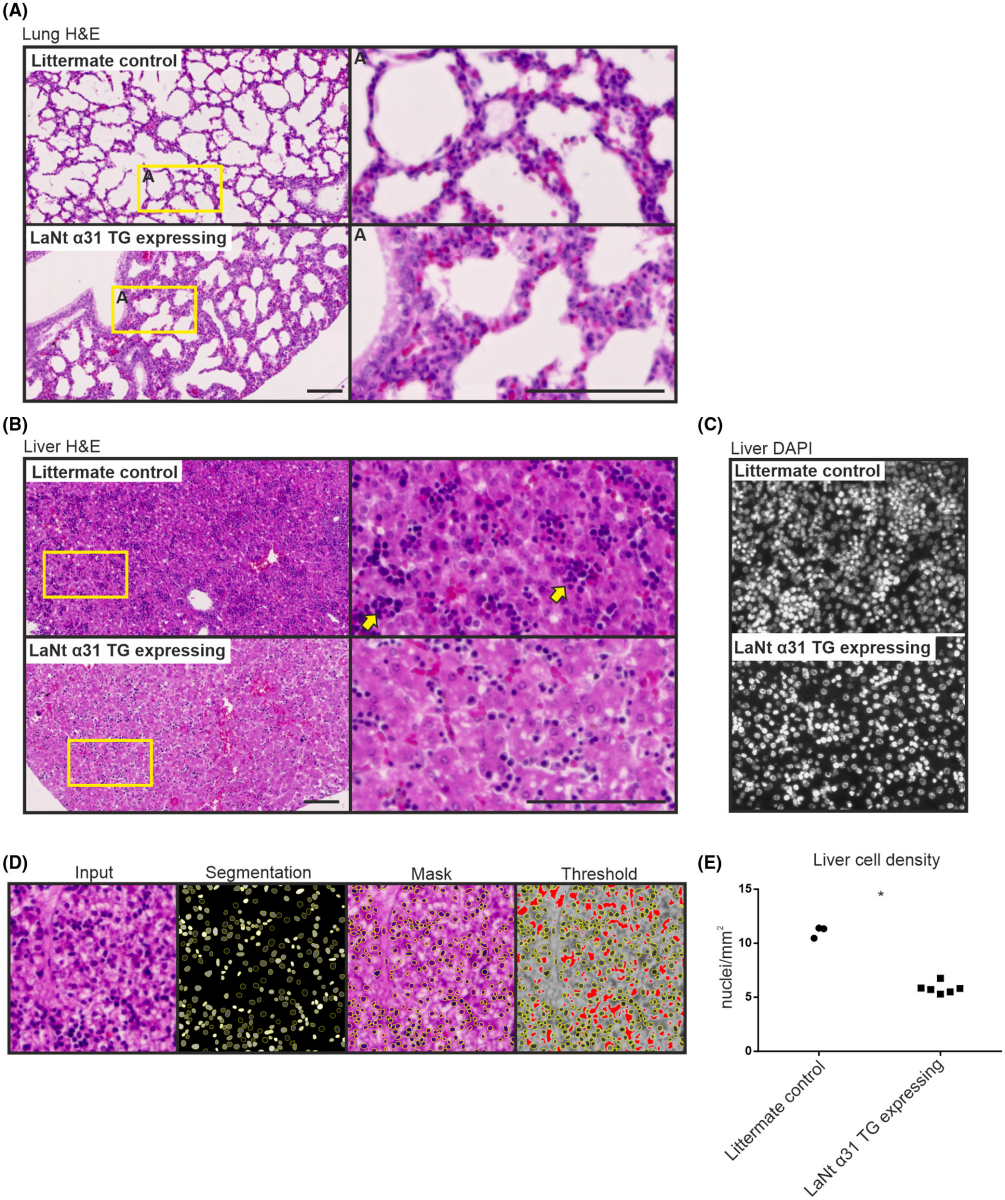
Mice expressing the LaNtα31 transgene display structural differences in the lung and a reduction of hematopoietic colonies in the liver. (A) H&E staining of FFPE sections (5 μm) of newborn UbCLaNtα31::R26CreERT2 transgenic mice lungs (A) and liver (B). Upper panel non‐expressing littermate controls, lower panels LaNt α31TG expressing animals. Right columns show increased magnification. Yellow arrowheads highlight areas of increased cell density. Scale bars =100 μm. (C) DAPI staining of littermate controls or LaNt α31 TG expressing mouse livers. (D) Representative image analysis method of determining nuclei count. (E) Quantification of nuclei. Each point represents the mean of the quantification of nuclei/mm^2^ from 2 separate microscope slides at different sectioning depths per mouse

Erythrocytes were present throughout the lung tissue and liver tissue of transgene expressing animals (Figure 6A,B). Structural differences were also apparent in the lungs, although it should be noted that the lungs of P0 mice were not inflated prior to fixation. Mice expressing LaNt α31 also displayed fewer, and less densely‐packed alveolar epithelial cells. The livers of mice expressing the LaNt α31 transgene exhibited a reduction in hematopoietic foci (Figure 6B,C). This reduction corresponded to a >33% reduction of total cell number (mean ± SD nuclei/mm^2^ littermate controls =11.0 ± 0.52, LaNt α31 expressing =5.8 ± 0.50, *p* =<.0001 determined by unpaired *t* test; Figure 6D,E). The bile ducts, sinusoid endothelium and hepatocyte morphology were histologically unchanged.

### 3.7 Keratin 14‐driven constitutive LaNt α31 induces a low offspring number

We generated an additional construct using the human keratin 14 (K14) promoter to drive expression of human LaNt α31, followed by a T2A element and a mCherry reporter (Figure S4A). The K14 promoter is expressed in the skin and the epithelia of tongue, mouth, forestomach, trachea, thymus and respiratory and urinary tracts,^67^, ^68^, ^69^ and has been described in the oocyte.^70^ The new construct was validated by transfecting into KERA 308 mouse epidermal keratinocytes and visualizing the mCherry fluorescence (Figure S4B) and immunoblotting for the LaNt α31 protein (Figure S4C). K14‐LaNtα31 transgenic mice were generated by pronuclear microinjection. However, unusually small litters were obtained from recipient CD1 mothers and mice containing the transgene DNA (Figure S4D) did not express the transgene at the protein level (Figure S4F,G). The unusually low offspring sizes, combined with the lack of protein expression in genotype‐positive mice suggests that expression of LaNt α31 under the control of the K14 promoter is lethal during development.

## 4 DISCUSSION

This study has demonstrated that LaNt α31 overexpression ubiquitously during development is lethal, causing widespread blood exudate throughout most tissues as well as changes to the tubules of the kidney and the basal layer of the epidermis, depletion of hematopoietic colonies in the liver, and evidence of capillary BM disruption. These findings build upon previous in vitro and ex vivo work that have implicated LaNt α31 in the regulation of cell adhesion, migration, and LM deposition.^24^, ^36^, ^39^ Importantly, they provide the first in vivo evidence that this little‐studied *LAMA3*‐derived splice isoform and newest member of the laminin superfamily has biological importance in BM and tissue formation during development and provide a valuable platform for onward investigation.

There are several plausible overlapping reasons that can explain the phenotype. As LM network assembly requires binding of an α, β, and γ LN domain,^14^, ^15^, ^16^, ^17^, ^71^ the presence of an αLN domain within LaNt α31 could influence LM‐LM interactions and therefore BM assembly or integrity. Indeed, LaNt α31, contains a perfect match to the LMα3b LN domain and biochemical assays have shown that the LMα3b LN domain is the most potent of the LM LN domains at disrupting LM111 polymerization in vitro.^72^ In vivo, the LaNt α31 protein would occupy βγ nodes but be unable to complete the polymer as a sheet‐like structure and, especially at locally high concentrations, would disrupt the laminin network. Consistent with this network disruption model, much of the LaNt α31 TG phenotype resemble those from mice where LM networks cannot form due to LN domain mutations. Specifically, whereas LMα5 knockout animals die at E17, LMα5 LN domain mutants were born at term with some animals surviving for weeks or months after birth. The LN domain mutant mice exhibited defective lung development and vascular abnormalities in the kidneys.^73^ Mice with LM β2 LN domain mutations or deletion of the LM β2 LN domain both exhibit renal defects, and although viable at birth, become progressively weaker and die between postnatal day 15 and 30.^74^, ^75^, ^76^, ^77^, ^78^, ^79^ In comparison with the LN‐domain specific mutant lines, the LaNt α31 TG expressing animals BM‐associated defects are somewhat similar although the overall effect is more severe and affects more tissues than each individual LN mutant line as anticipated by the more widespread expression of the transgene driven by UBC and R26 promoter activities.

Within the model of LaNt α31 inhibiting LM network assembly, there remains the question of how LaNt α31 influences tissues where the expressed LMs do not contain an α LN domain, and therefore are not able to polymerize.^16^ For example, The LM composition present within vessel BMs during development and lymph vessels is rich in the non‐polymerizing LM411.^80^, ^81^, ^82^ Here it should be noted that transgenic mice expressing the potent LM network disrupting protein netrin‐4 under the control of the K14 promoter were born smaller, redder, and with increased lymphatic permeability.^35^ In contrast, one might have anticipated that the LaNt α31 LN domain could compensate for the “missing” α LN domain in the vasculature and stabilize the weak, transient βγ LN dimers made by the LMβ1 and γ1 LN domains.^15^, ^17^, ^71^ However, the observed phenotype of blood exudate throughout the mouse tissues instead indicates that the LaNt α31 has a disruptive rather than stabilizing role.

It is also possible that the LaNt α31 transgene effects represent a signaling rather than structural role. Integrin‐mediated signaling from LaNt α31‐like proteolytically released LN‐domain containing fragments from LM α3b, α1, and β1 chains have been reported^29^, ^30^, ^31^ and some aspects of the UbCLaNt::R26CreERT2 phenotype are consistent with LaNt α31 acting in this way. For example one of the most striking phenotypes observed in the LaNt α31 transgenic mice was depletion of hematopoietic colonies in the liver, an essential stem cell niche during development.^83^, ^84^, ^85^ Integrins α6 and β1 are highly expressed in hematopoietic stem cells, and are central to the process of migration both in and out of the fetal liver.^86^, ^87^, ^88^ A netrin‐4/laminin γ1 complex has been shown to signal through the integrin α6β1 receptor to ERK1/2 and regulate neural stem cell proliferation and migration.^89^ LaNt α31 is also enriched in human and porcine limbal stem cell niche of adult corneas, with expression further upregulated upon ex vivo stem cell activation and wound repair.^36^ While these combined data suggest that direct signaling effects are possible with LaNt α31 binding to cell surface receptors, indirect effects are also probable. Altering LM network structural organization changes matrix stiffness, as has been demonstrated for netrin‐4,^90^ and also could influence outside‐in signaling through changing presentation of ligands or by modifying growth factor sequestration and release rates.^91^ Indeed, LM networks are also known to be critical for maintaining progenitor cell “stemness”.^92^, ^93^, ^94^, ^95^ Dissecting the direct versus indirect roles of LaNt α31 in intact tissue contexts is now a priority and the new transgenic mouse line provides a valuable resource to facilitate those onward investigations.

Moving forward, the role of LaNt α31 can now be determined in a tissue and context specific manner. Considering the widespread expression of LaNt α31,^37^ and the dramatic effects observed in this study, it is now important to determine effects in adult animals in normal conditions and following intervention and under lineage specific control. These studies should include tissues where no overt LaNt α31‐induced phenotype was observed. For example, although no muscle defects were observed in the animals in this study, LM network integrity is critical to muscle function, with the effects of LM α2 LN domain mutations or deletions developing muscular dystrophy and peripheral neuropathy with time^96^, ^97^, ^98^; therefore, longer‐term studies may reveal further phenotypes once tissues are placed under stress. Importantly, the biological function of LaNt α31 may be different at lower compared to higher concentration. At low concentrations, LaNt α31 may exploit the existing β‐γ LN domains to enable BM attachment in order to deliver a new receptor ligand to the BM. Therefore, a knockout model would also be valuable as well as further in vitro and biochemical analyses to dissect function.

Inherited disorders driven by variants to α LN domain have robustly established that LN domains are important for tissue function.^19^, ^22^, ^73^, ^99^, ^100^ The findings here add a new layer to this regulation. LaNt α31 is a naturally occurring protein generated from a laminin‐encoding gene via alternative splicing. These new results show that LaNt α31 is functional within a biological context. This is important as it raises the possibility of active regulation of LaNt α31 production via control of the splicing event as a mechanism to influence BM assembly/disassembly or matrix‐signaling by titrating LaNt α31 levels.^24^ Alternative splicing rates often change in normal situations during development and tissue remodeling, or in response to damage such as in wound repair, and are frequently dysregulated in pathological situations including frequently in cancer.^101^, ^102^, ^103^ Considered in this way, the finding the LaNt α31 is biologically active in vivo has exciting and far‐reaching implications for our understanding of BM biology.

## DISCLOSURES

The authors declare that there are no conflicts of interests.

## AUTHOR CONTRIBUTIONS

**Conor J. Sugden:** methodology, validation, formal analysis, investigation, data curation, writing – original draft, writing – review & editing, visualization. **Valentina Iorio:** methodology, investigation, data curation, writing – review & editing. **Lee D. Troughton:** methodology, writing – original draft, writing – review & editing. **Ke Liu:** methodology, writing – review & editing. **Mychel R. P. T. Morais**: methodology, formal analysis, writing – review & editing. **Rachel Lennon:** methodology, formal analysis, writing – review & editing. **George Bou‐Gharios:** conceptualization, methodology, writing – review & editing, supervision. **Kevin Hamill:** conceptualization, methodology, writing – original draft, writing – review & editing, supervision, funding acquisition.

## Supporting information

Fig S1

Fig S2

Fig S3

Fig S4

Text S1

## Data Availability

The data that support the findings of this study are available in the methods and/or supplementary material of this article. The transgenic mouse strains and plasmids are available on request from the corresponding author.
