# Supplementary figures and images for: Laminin N‐terminus α31 expression during development is lethal and causes widespread tissue‐specific defects in a transgenic mouse model
Source: FASEB J. 2022 Jun 1;36(7):e22318. doi: 10.1096/fj.202002588RRR (PMC9328196; doi:10.1096/fj.202002588RRR)

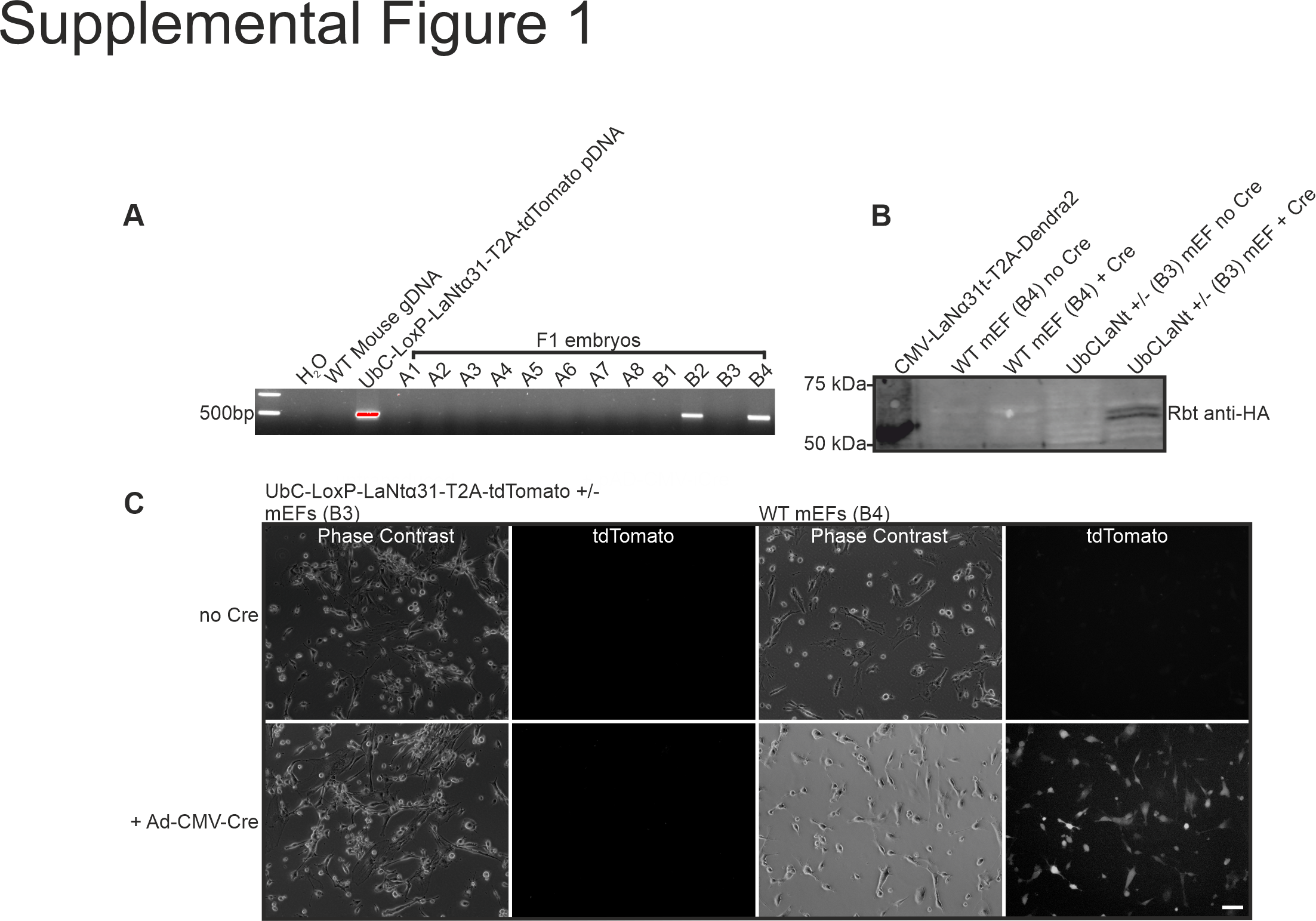

Supplement: Supplementary file 1 — Fig S1 [file FSB2-36-0-s005.tif]

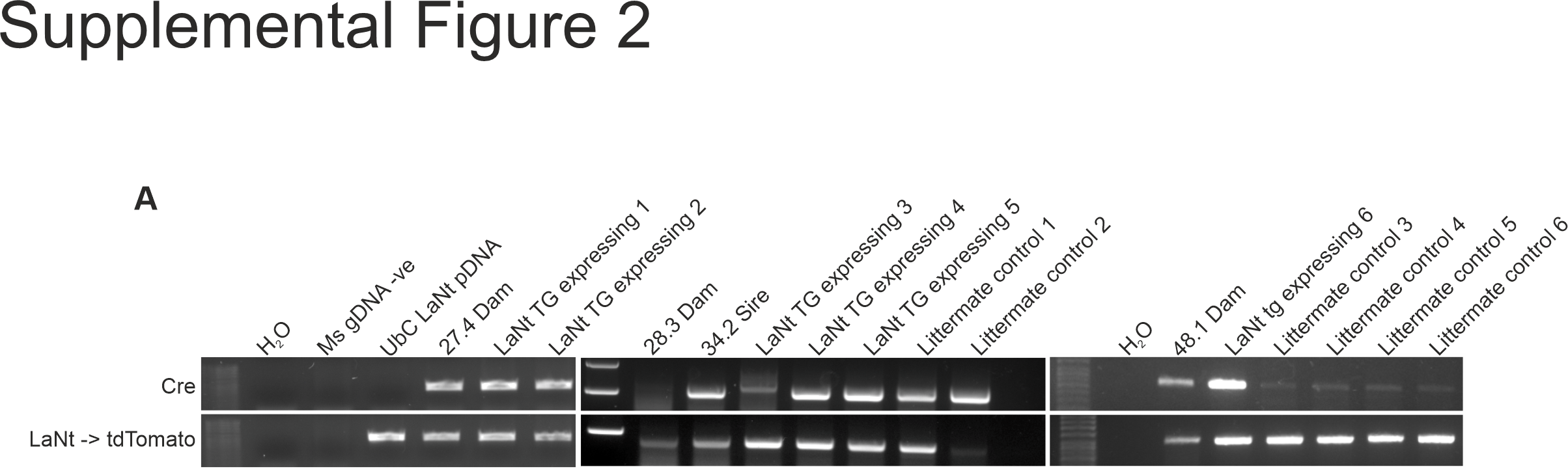

Supplement: Supplementary file 2 — Fig S2 [file FSB2-36-0-s002.tif]

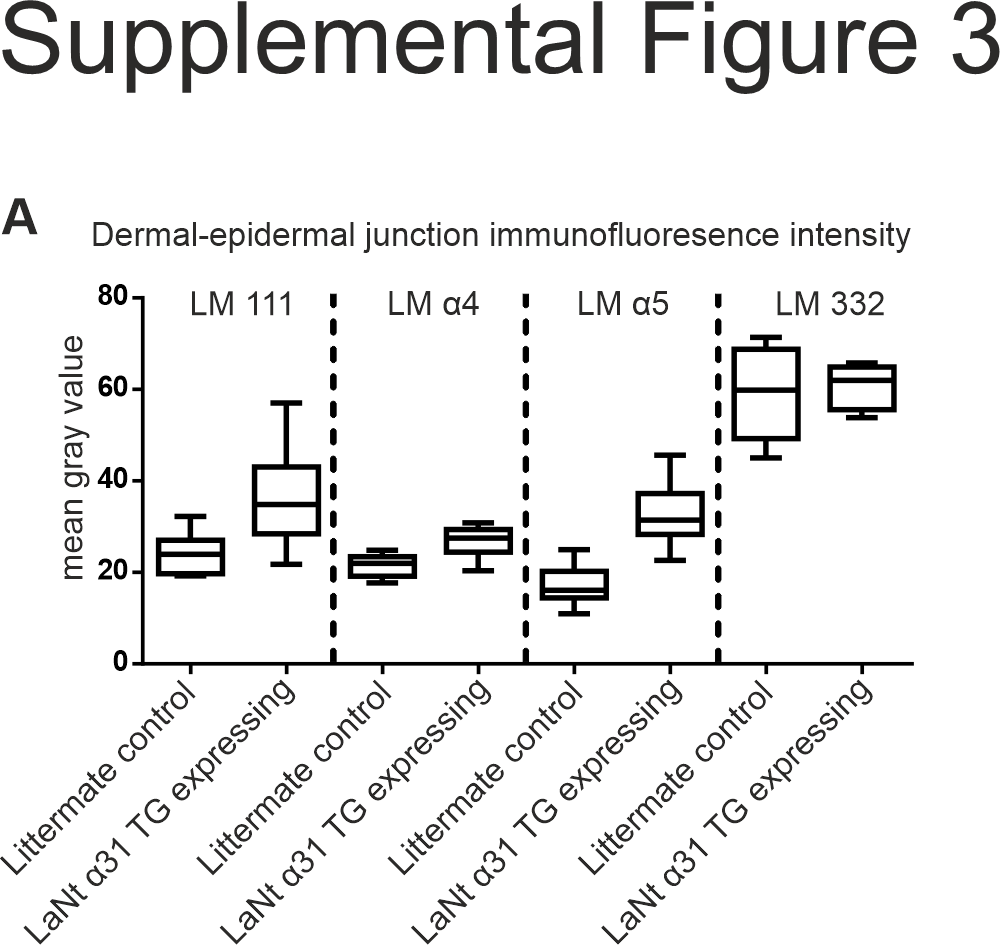

Supplement: Supplementary file 3 — Fig S3 [file FSB2-36-0-s001.tif]

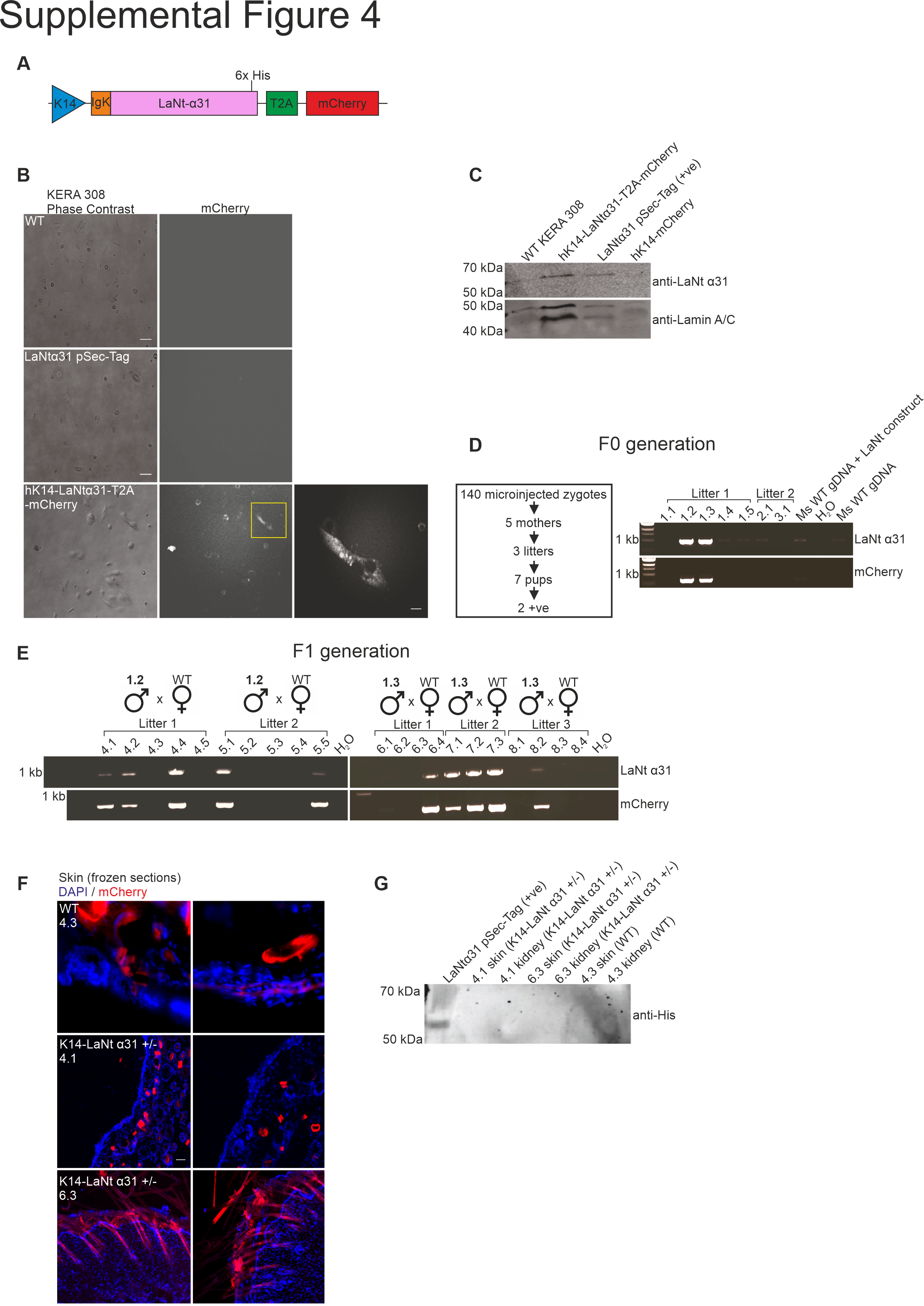

Supplement: Supplementary file 4 — Fig S4 [file FSB2-36-0-s003.tif]
