# Supplementary material for: Laminin N‐terminus α31 expression during development is lethal and causes widespread tissue‐specific defects in a transgenic mouse model
Source: FASEB J. 2022 Jun 1;36(7):e22318. doi: 10.1096/fj.202002588RRR (PMC9328196; doi:10.1096/fj.202002588RRR)
Supplement: Supplementary file 5 — Text S1 [file FSB2-36-0-s004.docx]

## Supplemental Methods

## Immunofluorescence Quantification

Images were processed using either Zen 2.6 (blue edition) (Zeiss) or ImageJ (National Institutes of Health, Bethesda, MD, United States)[^51^](#_ENREF_51). To quantify expression at the dermal-epidermal junction, a 4 mm x 6 mm selection was drawn around the dermal-epidermal junction and mean grey value measured using ImageJ per individual measurement.

## K14-LaNtα31 Transgenic Line Establishment

For K14-LaNtα31 transgenic mice, 140 embryos were transferred into five recipient CD1 mothers. Three small litters were born, totalling seven pups. Two pups possessed the transgene, and these were mated to generate F1 mice.

## hK14-LaNt α31

Full length LaNt α31 cDNA was amplified by PCR and inserted into pSecTag vector (Thermo Fisher Scientific), introducing Igκ leader sequence 5’ to the LaNt α31 sequence, and Myc and 6x His tags 3’ of the LaNt α31 sequence. The complete Igκ-LaNt α31-Myc-His sequence was inserted into pGEM®-5Zf(+) vector (Promega, Madison, WI) using NheI and PmeI (New England Biolabs), producing pGEM®-5Zf(+)-LaNt α31. Separately, the sequence encoding human keratin 14 (hk14) promoter was amplified by PCR, using primers introducing MluI 5’ and NdeI, NsiI 3’ of the sequence, and this was inserted into a bicistronic vector containing the mCherry seqeunce, producing phK14-mCherry. Finally, Igκ-LaNtα31-Myc-His was excised from pGEM®-5Zf(+)-LaNtα31 using NdeI and NsiI (New England Biolabs) and inserted into phK14-mCherry, to produce phK14-LaNtα31-T2A-mCherry.

## Supplemental Figure Legends

## Supplemental Figure 1 - UbC-LoxP-LaNt-α31-T2A-tdTomato embryonic fibroblast express the transgene upon transduction with a Cre recombinase-coding adenovirus

A) PCR amplicons produced using primers amplifying the UbC-LoxP-LaNt-α31-T2A-tdTomato transgene from gDNA of F1 UbC-LoxP-LaNt-α31-T2A-tdTomato embryos . B) Western blot of protein lysates from explanted F1 mouse embryonic fibroblasts processed with anti-HA antibodies. C) Fluorescence microscopy images of explanted cells from UbC-LoxP-LaNt-α31-T2A-tdTomato F1 mice. Scale bar = 100 µm.

## Supplemental Figure 2 – Genotyping for UbC-LoxP-LaNt-α31-T2A-tdTomato and R26CreERT2 transgenes in mice used in this study.

A) PCR amplicons produced using primers amplifying either the Cre recombinase gene or UbC-LoxP-LaNt-α31-T2A-tdTomato transgene on gDNA extracted from transgenic mouse embryos from the mating of UbCLaNtα31 x R26CreERT2 mice.

## Supplemental Figure 3 – Quantification of immunofluorescence intensity at the dermal-epidermal junctiom.

A) Mean gray intensity of immunofluorescence intensity of different anti-laminin antibodies at the dermal-epidermal junction. Each box and whisker plot represents at least ten individual measurements.

## Supplemental Figure 4 – Transgenic expression of LaNt α31 under control of the human keratin-14 promoter results in a low number of offspring.

A) Diagram of the phK14-LaNtα31-T2A-mCherry construct. B) Fluorescence microscopy images of KERA 308 cells transfected with phK14-LaNtα31-T2A-mCherry. Scale bar = 10 µm. C) Western blot of protein lysates from transfected KERA 308 cells. D) Schematic of F0 mice generation and PCR genotyping of F0 mice. E) PCR genotyping of F1 mice. F) Representative fluorescence images of frozen sections from F1 mice tissues. G) Western blot of tissue lysates from F1 mice, probed with anti-His antibodies.
